# The relationship between independent and dependent life events and depression symptoms in Sri Lanka: a twin and singleton study

**DOI:** 10.1007/s00127-019-01765-z

**Published:** 2019-09-03

**Authors:** Helena M. S. Zavos, Bethan Dalton, Kaushalya Jayaweera, Lisa Harber-Aschan, Gayani Pannala, Anushka Adikari, Stephani L. Hatch, Sisira Siribaddana, Athula Sumathipala, Matthew Hotopf, Frühling V. Rijsdijk

**Affiliations:** 1 Department of Psychology, Institute of Psychiatry, Psychology and Neuroscience, King’s College London, London, UK; 2 Section of Eating Disorders, Department of Psychological Medicine, Institute of Psychiatry, Psychology and Neuroscience, King’s College London, London, UK; 3 Institute for Research and Development, Colombo, Sri Lanka; 4 Psychological Medicine Department, Institute of Psychiatry, Psychology, and Neuroscience, King’s College London, London, UK; 5 Department of Medicine, Rajarata University of Sri Lanka, Anuradhapura, Sri Lanka; 6 School of Primary, Community and Social Care, Faculty of Medicine & Health Sciences, Keele University, Staffordshire, UK; 7 Social Genetic and Developmental Research Centre, Institute of Psychiatry, Psychology and Neuroscience, King’s College London, London, UK; 8 NIHR Biomedical Research Centre for Mental Health at the South London and Maudsley NHS Foundation Trust, King’s College London, London, UK

**Keywords:** Life events, Depression, Sri Lanka, Twins, Genetics

## Abstract

**Purpose:**

Life events have been associated with a variety of mental health conditions including depression. There is a scarcity of research in South Asia exploring the aetiology of independent and dependent life events and their relationship with depression symptoms. This study aimed, in a Sri Lankan population, to identify the socio-demographic correlates and genetic and environmental influences on independent and dependent life events and their relationship with depression.

**Methods:**

Questionnaire data came from the Colombo Twin and Singleton Follow-up Study, CoTaSS-2 (*N* = 3969), a population study of Sri Lankan twins and singletons. Lifetime-ever independent and dependent life events were measured using a questionnaire and depressive symptoms using the Revised Beck’s Depression Inventory. Structural Equation Model-fitting analyses explored the genetic and environmental influences on life events and depression.

**Results:**

Living in a rural environment and financial hardship were associated with greater reporting of independent and dependent life events. Sex differences were evident in the aetiology of life events and depression symptoms. Independent and dependent life events, but not depression symptoms, were heritable in males. Independent life events and depression symptoms, but not dependent life events, were heritable in females. Non-shared environmental influences explained phenotypic associations between independent life events and depression symptoms in both males and females. Genetic and non-shared environmental influences explained the phenotypic associations between dependent life events and depression symptoms in males. Only non-shared environment explained the covariation between dependent life events and depression symptoms in females.

**Conclusions:**

Socio-demographic correlates of independent and dependent life events were similar to those reported in Western populations. Life events were associated with increased depression symptoms. Contrary to research in Western populations, we found that non-shared environmental, rather than genetic, influences explained much of the covariation between life events and depression symptoms. This suggests that whilst independent LEs may be heritable, the relationship is unlikely to be confounded by genetic influences and has significant implications for possible interventions for depression.

**Electronic supplementary material:**

The online version of this article (10.1007/s00127-019-01765-z) contains supplementary material, which is available to authorized users.

## Introduction

Life events (LEs) are an important component in the development of mental health conditions including depression [1]. It is increasingly recognised that LEs, which can include both positive and negative events, are not simply passive experiences that happen to people, but are instead associated with a range of demographic, behavioural and genetic factors [2, 3]. Understanding the factors associated with exposure to LEs can be helpful in understanding their relationship with mental health conditions such as depression. However, despite the disease burden mental health conditions place on Low and Middle Income Countries (LMIC) [4], research into the role and aetiology of LEs in mental health conditions has been largely restricted to High Income Western populations. There is large variation in the frequency and exposure to LEs (e.g. disease, natural disasters) between LMIC and High Income Countries (HIC) [5]. The prevalence of depression has been shown to be different in HICs compared to LMICs, prevalence of lifetime depression higher in HICs [6]. It is, therefore, possible that a different pattern of effects will be evident in Sri Lanka, a LMIC in South Asia, the setting of the current study.

Research in high-income settings has shown that a range of demographic factors, including minority ethnicity, younger age and lower social economic status, are associated with reporting of LEs [2]. Studies have shown that socio-economic disadvantage (e.g. low income and low occupational status) have been associated with greater reporting of LEs [2, 7, 8]. This may be due to the limited opportunities and resources that are associated with lower income status, which puts people at greater risk of LEs. Given the increased levels of economic disadvantage in LMIC compared to HIC, it is important to understand the link with LEs in this different environmental and cultural context. Understanding whether certain demographic groups are more likely to experience LEs can help guide prevention efforts [2].

Genetically sensitive designs can also be used to understand the non-random distribution of LEs, and the relationship between LEs and depression. If a genetic influence is observed on an environment exposure, it is known as ‘gene-environment correlation’ (rGE) and points to the fact that a person’s behavior, which is genetically influenced, can make one more likely to experience LEs. There are three main ways that genes are thought to influence the environment [9]; (1) passive rGE (association between the genotype a child inherits from their parents and the family environment that they are brought up in), (2) evocative rGE (association between an individual’s genetically influenced behaviour and other people’s reaction to it) and (3) active rGE (when individuals actively select, create and modify their environmental experiences based on their genetically mediated dispositions). Research has provided evidence for a genetic influence on reporting of LEs [3, 10, 11], however, some have not supported such associations [12].

As exposure to LEs can be associated with an individual’s behavior, the causal relationship between LEs and depression has been hard to establish. Previous studies have distinguished between ‘independent’ LEs and ‘dependent’ LEs [1]. Independent LEs are defined as not being associated with the individuals behaviour or current mental health, for example experiencing a natural disaster. ‘Dependent’ LEs are events which may be associated with an individual’s behaviour or psychopathology, for example a relationship breakdown [1]. One benefit of distinguishing between independent and dependent LEs is that the direction of effects for independent LEs is clearer as they are unlikely to be confounded by individuals’ personality or current mood state. Independent LEs have been significantly associated with the onset of depression [13–16] suggesting LEs do have a causal role in the development of depression. Twin and molecular genetic studies that have considered independent and dependent LEs separately have tended to show higher heritability for dependent LEs compared to independent LEs [17–19], although findings have been mixed [11, 20].

Genetically sensitive designs can also be used to understand whether similar genetic or environmental influences are implicated in the relationship between LEs and depression. Studies looking at the aetiological relationship between LEs and depression have tended to show that shared genes account for some of the relationship between them [21, 22]. This suggests that a genetically influenced set of traits (e.g. a personality trait) increases an individual’s likelihood of selecting themselves into environments associated with LEs and increases their vulnerability to developing depression [13].

Whether the same LEs operate equivalently in different settings has not been well established due to the paucity of research in culturally diverse environments [23]. One study looking at the aetiology of LEs in Sri Lanka, using data from a previous wave of the current sample, showed that the variance in the reporting of LEs was explained by additive genetics (44%) and non-shared environmental influences equally (53%) [24]. However, only six dependent LEs were considered compared to the 19 independent and 22 dependent LEs considered in the current study. Moreover, the previous study was not able to examine the aetiological overlap between LEs and depression. Evidence of a genetic link between LEs and depression requires further exploration in non-Western populations, particularly given that the aetiology of depression in Sri Lanka, South Korea and China is different to Western populations [25–27], with men showing low heritability and women showing moderate heritability in both Sri Lanka and South Korea.

The current study sought to investigate in Sri Lanka, (1) the socio-demographic correlates of independent and dependent lifetime-ever LEs to test the hypothesis that a range of socio-demographic factors are associated with experiencing one or more LEs; (2) the aetiology of lifetime-ever independent and dependent LEs; and (3) whether the genetic and environmental influences that act on LEs are the same as those that influence depression symptoms. In line with previous research in Western populations we expect that LEs will be heritable, with greater heritability shown for dependent LEs. Moreover, we predict that the genetic influences on LEs will be correlated with depression.

## Methods

COTASS-2 took place between 2012 and 2015, and is a follow-up study of the Colombo Twin and Singleton Study (COTASS-1), conducted in 2005–2007 [28]. In COTASS-2, questionnaire data was available from 3934 twins and singletons (Twin *N* = 2899, Singleton *N* = 1035), 76.4% of the original COTASS-1 sample. Number of individuals by zygosity is given in eTable 1. Twins and singletons differed on a number of socio-demographic characteristics. Singletons for example had lower socio-economic status, reported lower education and greater financial strain. Singletons were also more likely to be older, female, and Sinhalese ethnicity [29]. Full details of the COTASS-2 study are described in Jayaweera et al. [29]. Ethical approval for the study was received from the Faculty of Medical Sciences University of Sri Jayewardenepura Ethical Review Committee (USJP ERC) (reference number: 596/11) and from the Psychiatry, Nursing and Midwifery Research Ethics Subcommittee, King’s College London, UK (reference number: PNM/10/11-124).

### Interview measures

*Sociodemographic characteristics:* Sociodemographic information was collected through measures adapted from the 2012 Sri Lankan census. Measures included sex, age, ethnicity, occupation and education. To understand participants economic status participants were asked ‘how well do you feel you are managing financially these days?’. Responses were on a five-point scale ranging from ‘living comfortably’ to ‘finding it very difficult to make ends meet’.

*Life events:* Lifetime-ever LEs were measured using a 56-item questionnaire which was based on the list of threatening experiences [30] but was culturally adapted for the Sri Lankan population by AS and SS (authors of the current study). For example, items such as ‘trouble with in laws’ and ‘no money for food, education, health or other essential things in life’ were included. Participants were asked to indicate whether they had ever experienced any of the LEs (response: yes/no). Events were classified as independent or dependent. This distinction was made according to whether the event is likely to arise from an individual’s behavior; thus ‘spouse/girlfriend/boyfriend died’ is an example of an independent event, whereas ‘took on greatly increase workload’ is an example of a dependent event. Categorization of independence/dependence were made by three researchers (HZ, BD and FR) and inconsistencies resolved by discussion. Items that were could not be distinguished as either dependent or independent, even after discussion, were not included. Nineteen items were designated as independent and 22 as dependent (see Table 1). Both scales demonstrated adequate internal consistency (see Table 2).

**Table 1:** List of independent and dependent life events and number of times reported

List of independent and dependent life events	*N* (%)
**Independent life events**	
Spouse/girlfriend/boyfriend died	211 (5.37)
*Family member other than spouse or child dies*	*1973 (50.17)*
Child died	240 (6.13)
Pet died	1050 (26.72)
Close friend died	971 (24.71)
*Child, spouse or close member of the family had problems in school, university or other training*	*438 (11.15)*
*Child, spouse or close member of the family cannot find a job*	*547 (13.92)*
Miscarriage or still birth	582 (14.90)
Found that cannot have children	139 (3.56)
Unable to get treatment for an illness or injury	179 (4.56)
Started menopause	72 (1.85)
Have a child with special needs (medical, mental or educational)	65 (1.66)
*Illness, injury or accident of spouse, child parent or close member of the family*	*1367 (34.79)*
*Close relative or friend suffered a serious illness or accident*	*1109 (28.20)*
Experienced a natural disaster	531 (13.50)
Sexual assault forced or pressured sexual contact	50 (1.27)
Scolded or criticised unfairly by superior at school or at work	560 (14.24)
Felt that you were mistreated because of your religion or ethic group	65 (1.65)
Lost a home through fire, flood or other disaster	223 (5.67)
**Dependent life events**	
Changed school, university or training program	536 (13.63)
Had problems, poor results or failure at school/university/training program	732 (18.61)
Had trouble with employer (e.g. in danger of losing job, being suspended or demoted)	502 (12.76)
Took on a greatly increased workload	817 (20.77)
Changed jobs for one that was worse or no better than the previous one	117 (2.97)
Could not find a job	617 (15.69)
Could not find university, or a school or program	153 (3.89)
Love relationship ended (including an engagement)	914 (23.23)
Relationship with spouse/significant other changed for the worse, without separation or divorce	388 (9.90)
Trouble with in-laws	565 (14.42)
*Serious family argument other than with spouse*	*702 (17.85)*
Became pregnant unexpectedly (may be out of wedlock)	132 (3.38)
Birth of a second or later child	494 (12.89)
Problems with the police involving court appearance	702 (17.85)
Inability to pay a loan	688 (17.50)
Suffered a financial property or business loss	617 (15.69)
Confiscation of an item due to inability to pay a loan	160 (4.07)
Moved to a worse (not better) residence or neighborhood	355 (9.03)
Took out a loan (mortgage)	471 (11.98)
Lost a drivers license, national identity card or a valuable document (deed)	463 (11.77)
No money for food, education, health and other essential things in life	1153 (29.31)
Victim of a financial scam or a swindler	649 (16.50)

Italicised events indicate events which could be obligatory shared between members of twin pair. N = number of participants reporting each event

**Table 2:** Descriptive statistics for independent life events, dependent life events and depression symptoms

	Independent life events *N* (%)	Dependent life events *N* (%)	
*N* reported events			
0	708 (18.02)	791 (20.13)	
1	746 (18.99)	679 (17.28)	
2	664 (16.90)	636 (16.19)	
3	573 (14.58)	456 (11.61)	
4	450 (11.45)	377 (9.60)	
5	355 (9.04)	284 (7.23)	
6+	433 (11.02)	706 (17.97)	

	Independent life events	Dependent life events	BDI-II depression symptoms
Mean (SD)	3.03 (2.89)	2.64 (2.18)	4.86 (6.19)
Range	0–12	0–21	0–53
Skew	0.80	1.25	2.28
Cronbach’s *α*	0.61	0.73	0.62

After transformation and age and sex regression the skew statistics for Independent life event, dependent life events and depression symptoms were 0.22, 0.48 and 0.29, respectively

*SD* standard deviation

*Depression symptoms:* The Revised Beck Depression Inventory (BDI-II) was used to measure depressive symptom severity in the past 2 weeks [31]. The BDI-II is a self-report questionnaire consisting of 21 items. Each item consists of four statements arranged in increasing severity corresponding to a particular symptom of depression; the statements are scored on a 4-point scale (0–3). The score for each item is summed to create a single score. Higher total scores indicate a greater severity of depression symptoms. The timeframe for the response is 2 weeks. The BDI-II is a reliable and valid measure of depression [32, 33] and showed acceptable internal consistency in the current sample (*α* = 0.62; see Table 2).

*Zygosity:* Zygosity was ascertained in CoTaSS-1 using a questionnaire measure of similarity [28]. If zygosity was missing in CoTaSS-1, it was replaced with zygosity information collected using the same questionnaire in CoTaSS-2 (*n* = 88).

### Statistical analyses

All analyses were performed using statistical packages STATA 14 [34] and Open MX [35]. Open MX uses the method of maximum likelihood estimation and is widely used for analysing genetically sensitive data. The relationship between LEs and depression symptoms was assessed using linear regression with depression symptoms as the outcome variable. Analyses were clustered using the “cluster” command in STATA, this returns clustered standard errors and accounts for the non-independence of twins in the sample.

In line with standard behavioural genetics procedures, the effects of sex and age were regressed out, and analyses were conducted using residuals [36]. Scales for LEs and depression symptoms were transformed using square root transformation techniques to reduce skew and to ensure that the assumption of having a normal distribution was met for genetic modelling (see Table 2).

### The twin design

The twin design uses data collected from monozygotic (MZ) and dizygotic (DZ) twin pairs to estimate the extent to which variations in a single phenotype, or covariation between phenotypes are explained by genetic or environmental influences. The twin method is based on the following assumptions: (1) MZ twin pairs share 100% of their genes and DZ twin pairs share on average 50% of their segregating genes; (2) MZ and DZ twin pairs share environmental factors common to both twins in the same family (‘shared environment’); and (3) MZ and DZ twin pairs differ from one another due to exposure to environmental factors which are specific to the individual (‘non-shared environment’). Correlations between MZ twin pairs and DZ twin pairs can then be used to establish the role of genetic and environmental factors. If, for example, MZ twins are more correlated on a particular trait than DZ twins then genetic influences are assumed. Shared environmental influences are indicated if the DZ twin correlation is more than half of MZ twin pairs. Lastly, the extent to which MZ twins differ is due to non-shared environmental influences, this component also include measurement error [37].

Structural equation modelling techniques which used raw data maximum likelihood were employed to establish the relative importance of additive genetic (*A*), common environment (*C*) and non-shared environmental influences (*E*) contributing to a phenotype [37]. First a heterogeneity ACE model was run. This model estimates *A*, *C* and *E* separately for males and females allowing for quantitative difference in males and females. A homogenetity model in which *A*, *C* and *E* are equated in males and females was then run and the fit compared to the heterogeneity model.

This technique further extends to bivariate analyses, by exploring the covariation between phenotypes. The extent to which the genetic and environmental influences are correlated between phenotypes is calculated by estimating the genetic correlations (*r*^a^), shared environment correlations (*r*^c^) and non-shared environment correlations (*r*^e^) [37]. Specification of the bivariate model was in line with Neale, Roysamb and Jacobson [38], this model involves using a correlation approach to ensure that the order of the variables does not affect the ability of the model to account for the dizygotic opposite sex (DZOS) data thus allowing for the inclusion of opposite sex pairs. A heterogeneity model was first fit to the data followed by a homogeneity model. The difference in fit of these models were evaluated by likelihood ratio testing.

Fit statistics provided by Open MX for raw data modelling is minus twice the log likelihood (−2LL) of the observations. All confidence intervals of parameter estimates were obtained by maximum likelihood.

## Results

The type and number of independent and dependent LEs experienced by individuals in the sample are given in Tables 1 and 2. The mean number of lifetime-ever independent LEs was 3.03: 18% of the sample did not experience any independent LEs, 46% reported 3 or more independent LEs and 11% reported 6 or more LEs. The most commonly experienced independent LEs were ‘family member other than spouse or child dies’ (reported by 50%), ‘illness or injury of close family member’ (reported by 35%) and ‘serious injury or illness of close relative or friend’ (reported by 28%). Females reported more independent LEs than males (*β* = 0.26 (0.11–0.40), *p* < 0.01).

For dependent lifetime-ever LEs, the mean reported LEs was 2.64: 20% reported none, 47% reported 3 -or more and 18% reported 6 or more. The most commonly reported lifetime-ever dependent LEs were ‘no money for essential things in life’ (reported by 29%), ‘love relationship ended’ (reported by 23%) and ‘took on greatly increased workload’ (reported by 21%). Males reported more dependent LEs compared to females (*β* = −0.25 (−0.45/−0.05), *p* = 0.01).

The mean score of BDI-II depression symptoms reported in the current sample was 4.86 (see Table 2). Higher depression scores were observed in females compared to males (*β* = 1.46 (1.07–1.86), *p* < 0.01). When categorised, 9% of the sample scored 14 or more on the BDI-II, which indicates at least mild depression. A similar sex effect was observed for mild depression, with females reporting mild depression significantly more than males (*χ*^2^ = 21.31, *p* < 0.01).

### Socio-demographic factors associated with reporting of independent and dependent LEs

Adjusted, and unadjusted, associations between independent and dependent LEs and a number of socio-demographic factors are shown in Table 3. Those who had been previously married reported a greater number of all types of LEs and these associations survived adjustment for other socio-demographic correlates. Education was not strongly associated with reporting of LEs. However, individuals who had university education reported fewer dependent LEs. Living in a more rural environment was associated with higher reporting of LEs. Higher levels of financial strain were associated with greater reporting of independent and dependent LEs and this remained significant after adjustment for other socio-demographic factors.

**Table 3:** Associations between independent and dependent life events and socio-demographic characteristics

	Independent LEs	Dependent LEs	Independent LEs	Dependent LEs
	Unadjusted *β*		Adjusted *β*	
*Sex*				
Male (ref)				
Female	0.12 (0.05/0.18)**	− 0.09 (− 0.16/− 0.02)*	0.03 (− 0.03/0.10)	− 0.17 (− 0.23/− 0.10)**
*Twin status*				
Singleton (ref)				
Twin	− 0.44 (− 0.51/− 0.36)**	− 0.20 (− 0.28/− 0.12)**	− 0.37 (− 0.45/− 0.30)**	− 0.27 (− 0.34/− 0.19)**
*Age*				
19–29 (ref)				
30–39	0.15 (0.05/0.25)**	0.17 (0.07/0.27)**	0.05 (− 0.06/0.15)	0.07 (− 0.04/0.18)
40–49	0.31 (0.21/0.41) **	0.23 (0.12/0.33)**	0.15 (0.04/0.26)*	0.07 (− 0.04/0.19)
50–59	0.40 (0.30/0.51) **	0.09 (− 0.02/0.20)	0.22 (0.10/0.34)**	− 0.08 (− 0.20/0.04)
60–69	0.31 (0.18/0.44) **	− 0.09 (− 0.21/0.03)	− 0.02 (− 0.16/0.12)	− 0.39 (− 0.53/− 0.24)**
70+	0.43 (0.28/0.59) **	− 0.21 (− 0.34/− 0.08)**	− 0.01 (− 0.18/0.17)	− 0.56 (− 0.72/0.40)**
*Ethnicity*				
Sinhala (ref)				
Tamil	− 0.05 (− 0.28/0.18)	0.11 (− 0.13/0.34)	0.03 (− 0.18/0.25)	0.06 (− 0.16/0.27)
Muslim	− 0.11 (− 0.27/0.05)	− 0.09 (− 0.26/0.07)	0.02 (− 0.14/0.18)	− 0.06 (− 0.23/0.11)
Other Minority	− 0.53 (− 0.84/− 0.22)**	− 0.31 (− 0.71/0.08)	− 0.60 (− 0.91/− 0.29)	− 0.36 (− 0.69/− 0.02)*
*Marital status*				
Married (ref)				
Previously married	0.43 (0.31/0.54) **	0.12 (0.00/0.24)	0.33 (0.20/0.45)**	0.25 (0.13/0.37)**
Never married	− 0.27 (− 0.35/− 0.20)**	− 0.14 (− 0.22/0.07)**	− 0.12 (− 0.21/− 0.02)*	− 0.11 (− 0.21/− 0.02)*
*Education*				
No education (ref)				
Grade 1–5	− 0.03 (− 0.39/0.33)	− 0.04 (− 0.42/0.34)	0.01 (− 0.32/0.31)	0.07 (− 0.04/0.18)
Grade 6 O/Ls	− 0.24 (− 0.57/0.10)	− 0.14 (− 0.50/0.22)	− 0.12 (− 0.42/19)	0.07 (− 0.04/0.19)
Passed O/Ls	− 0.29 (− 0.63/0.06)	− 0.30 (− 0.67/0.07)	− 0.06 (− 0.37− 0.24)	− 0.08 (− 0.20/0.04)
Up to/passed A/Ls	− 0.33 (− 0.67/0.02)	− 0.24 (− 0.61/0.13)	− 0.07 (− 0.37/0.24)	− 0.39 (− 0.53/− 0.25)**
University or higher	− 0.37 (− 0.73/− 0.01)*	− 0.23 (− 0.61/0.15)	− 0.06 (− 0.38/0.26)	− 0.56 (− 0.72/− 0.40)**
*Urbanicity*				
Urban (ref)				
Rural	0.39 (0.29/0.50)**	0.27 (0.16/0.38)**	0.42 (0.33/0.31)**	0.31 (0.20/0.42)**
Mixed	0.18 (0.09/0.26)**	− 0.01 (− 0.09/0.07)	0.17 (0.09/0.26) **	− 0.01 (− 0.09/0.06)
Outside Colombo	0.31 (0.17/0.44)**	0.30 (0.14/0.46)**	0.38 (0.24/0.52) **	0.34 (0.18/0.50)**
*Financial Strain*				
Living comfortably/doing alright (ref)				
Just about getting by	0.06 (− 0.03/0.15)	0.22 (0.23/0.32)**	0.02 (− 0.06/0.11)	0.22 (0.12/0.32)**
Difficult to make ends meet	0.28 (0.15/0.41)**	0.43 (0.30/0.57)**	0.23 (0.11/0.35) **	0.42 (0.28/0.55)**
Very difficult to make ends meet	0.43 (0.24/0.62)**	0.80 (0.56/1.04)**	0.38 (0.19/0.57) **	0.80 (0.57− 1.03)**

Regression conducted using standardised outcome variables. Adjusted *β* coefficients were calculated after included all other socio-demographic variables in the table

*O/Ls* O-levels, *A/Ls* A-levels

*p < 0.05; **p < 0.01

### Genetic and environmental influences associated with independent LEs, dependent LEs and depression symptoms

All ACE models fitted the constrained saturated model well (see appendix eTable 2).

Independent LEs: The similar correlations in both MZ males and females and DZ males and females did not indicate sex differences (see Table 4) and this was supported by model fitting results. Variance in independent LEs was explained by significant genetic influences (24% 95% CIs: 0.03–0.42) and non-shared environmental influences (65% 95% CIs: 0.58–0.72).

**Table 4:** Twin correlations and univariate ACE estimates for independent life events, dependent life events and depression

	Independent life events	Dependent life events	Depression
MZM	0.34 (0.22–0.44)	0.45 (0.34–0.53)	0.30 (0.15–0.42)
DZM	0.22 (0.06–0.36)	0.28 (0.11–0.42)	0.25 (0.09–0.39)
MZF	0.36 (0.26–0.45)	0.31 (0.21–0.40)	0.35 (0.24–0.45)
DZF	0.24 (0.10–0.36)	0.31 (0.18–0.42)	0.21 (0.07–0.34)
DZOS	0.24 (0.12–0.35)	0.25 (0.15–0.35)	0.11 (0.00–0.22)

	*A*	*C*	*E*
Independent life events	0.24 (0.03–0.42)	0.11 (0.00–0.28)	0.65 (0.58–0.72)
Dependent life events			
Male	0.30 (0.01–0.49)	0.14 (0.01–0.39)	0.56 (0.47–0.66)
Female	0.03 (0.00–0.32)	0.29 (0.04–0.38)	0.68 (0.60–0.76)
Depression			
Male	0.05 (0.00–0.42)	0.24 (0.00–0.37)	0.71 (0.59–0.83)
Female	0.24 (0.00–0.45)	0.10 (0.00–0.33)	0.65 (0.55–0.76)

*MZM* monozygotic male, *DZM* dizygotic male, *MZF* monozygotic female, *DZF* dizygotic female, *DZOS* dizygotic opposite sex, *A* additive genetic influences, *C* shared environmental influences, *E* non-shared environmental influences

Dependent LEs: The best-fitting model was the quantitative heterogeneity ACE, which indicated sex differences. Genetic influences were implicated in males (30% 95% CIs: 0.01–0.49 of variance explained) but not females (3% 95% CIs: 0.00–0.32 of variance explained). Significant shared and non-shared environmental influences were indicated in both males and females.

Depression symptoms: The best-fitting model was the quantitative heterogeneity ACE model, suggesting sex differences. For males, the variance was explained by a 5% (95% CIs: 0.00–0.42) additive genetics, 24% (95% CIs: 0.00–0.37) shared environment and 71% (95% CIs: 0.59–0.83) non-shared environment. However, only non-shared environmental influences were significant. For females, 24% (95% CIs: 0.00–0.45) of the variance resulted from additive genetics and 65% (95% CIs: 0.55–0.76) by non-shared environment, with a small contribution of shared environment (10% 95% CIs: 0.00–0.33).

### Relationship between LEs and depression symptoms

*Phenotypic associations between LEs and depression*. Non-overlapping confidence intervals indicate that a significantly stronger correlation was observed between dependent LEs (Males rPh = 0.43 95% CI: 0.39–0.47; Female rPh = 0.42 95% CI: 0.39–0.46) and depression compared to independent LEs (Males rPh = 0.33 95% CI: 0.29–0.38; Female rPh = 0.30 95% CI: 0.26–0.34).

### Genetic and environmental associations between LEs and depression symptoms

**Fig. 1:**
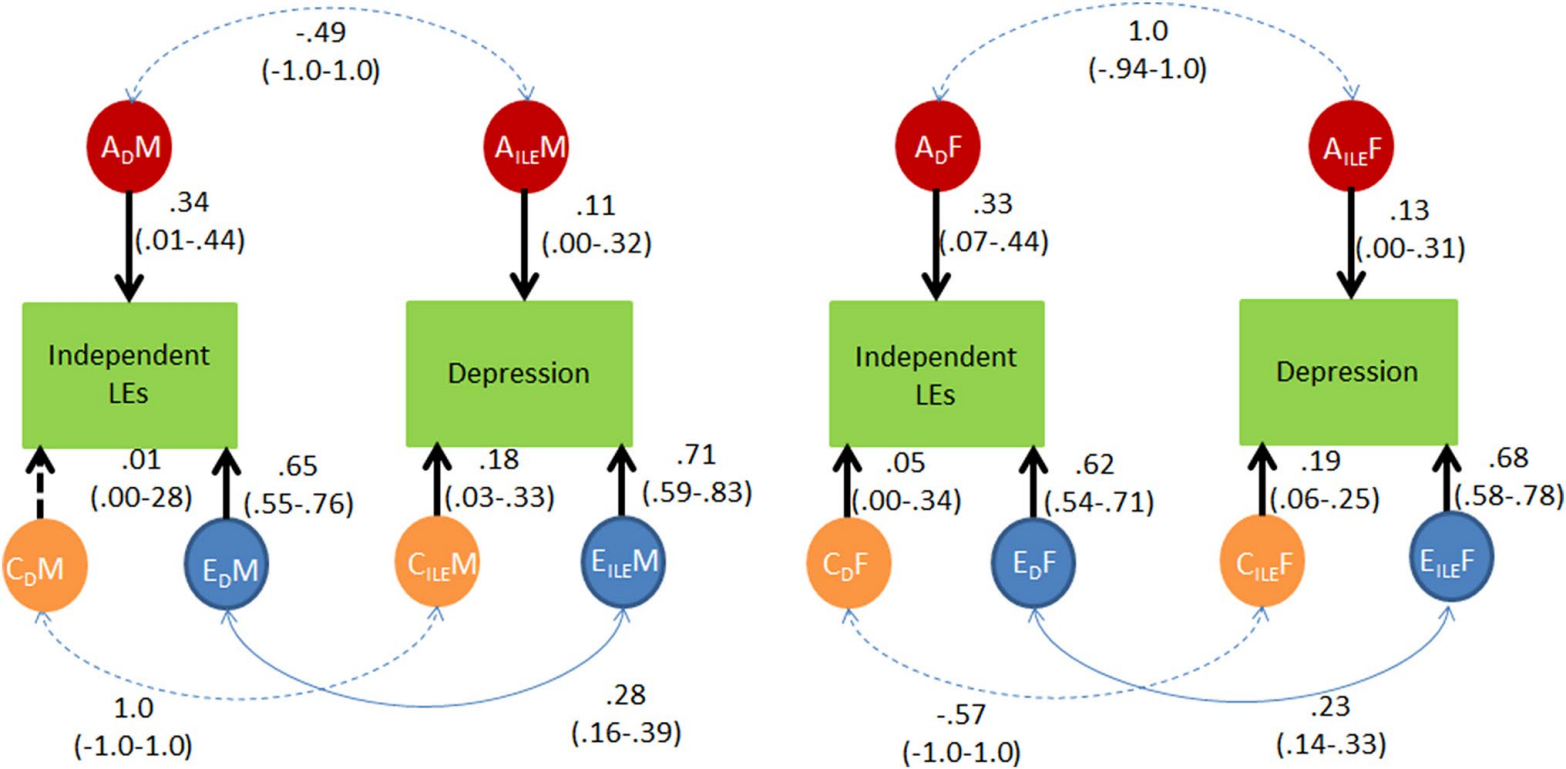
Genetic and environmental correlations between independent life events and depression. Results for males are shown on the left hand slide and for females on the right hand side of the figure. *A* = Additive genetic influences, *C* = Shared environmental influences, *E* = non-shared environmental influences; *M* = Male; *F* = Female. Discontinuous lines indicate non-significant effects, continuous lines indicate significant effects

*Independent LEs and depression.* The best fitting model, the quantitative heterogeneity model, allowed for sex differences between males and females for the parameter estimates as indicated by the univariate twin analyses (see appendix Table e3). Results of this analysis are shown in Fig. 1. In males, only non-shared environmental influences between depression symptoms and independent LEs were significant (*Re *= 0.28). This was similar in females where a moderate correlation between non-shared environment was also observed (*Re *= 0.23).

**Fig. 2:**
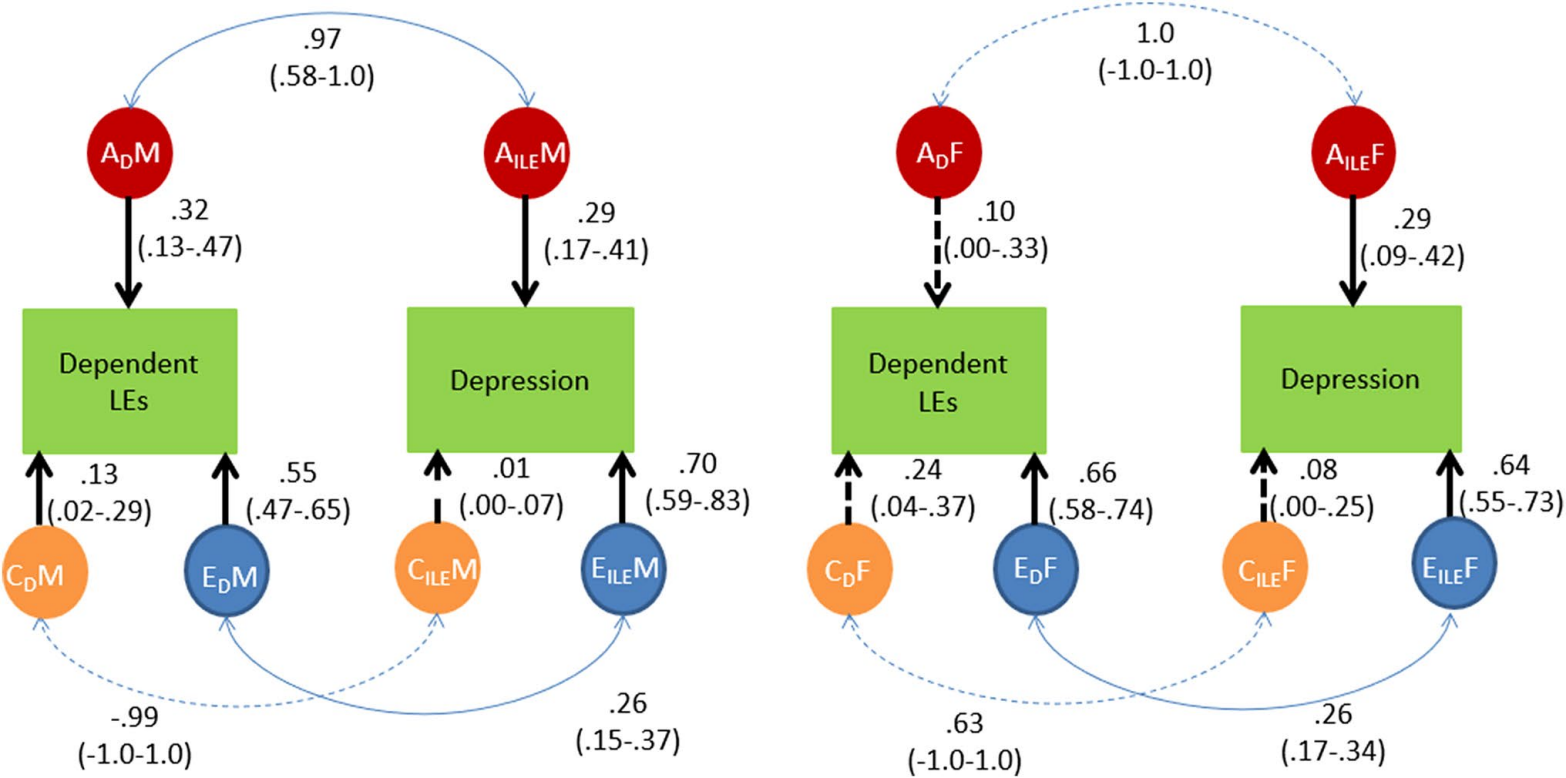
Genetic and environmental correlations between dependent life events and depression. Results for males are shown on the left hand slide and for females on the right hand side of the figure. *A* = Additive genetic influences, *C* = Shared environmental influences, *E* = non-shared environmental influences; *M* = Male; *F* = Female. Discontinuous lines indicate non-significant effects, continuous lines indicate significant effects

*Dependent LEs and depression symptoms.* Cross-twin cross-trait correlations indicating possible genetic factors in the relationship between dependent LEs and depression (see Table 5), the correlation between genetic factors were significant in males only (*Re* = 0.97; see Fig. 2). In both males and females, non-shared environmental influences were correlated (*Re* = 0.26 and 0.26, respectively).

**Table 5:** Phenotypic correlations and cross-twin cross trait correlations

	Depression-independent LEs	Depression- dependent LEs
rPh male	0.33 (0.29–0.38)	0.43 (0.39–0.47)
rPh female	0.30 (0.26–0.34)	0.42 (0.39–0.46)
MZM	0.13 (0.03–0.22)	0.27 (0.17–0.35)
DZM	0.15 (0.04–0.26)	0.14 (0.01–0.25)
MZF	0.16 (0.08–0.23)	0.26 (0.19–0.33)
DZF	0.03 (− 0.08–0.13)	0.15 (0.05–0.25)
DZOS	0.06 (− 0.03–0.14)	0.07 (− 0.01–0.16)

*rPh Male* phenotypic correlation males, *rPh female* phenotypic correlation females, *MZM* monozygotic male, *DZM* Dizygotic male, *MZF* monozygotic female, *DZF* dizygotic female, *DZOS* dizygotic opposite sex

## Discussion

This is the first study to investigate the socio-demographic correlates and genetic aetiology of independent and dependent LEs and depression symptoms in a non-Western population. Our findings regarding the socio-demographic association with LEs were largely in line with our predictions. A number of socio-demographic factors were associated with increased reporting of LEs, including younger age and marital status. Having high levels of financial strain was particularly indicative of reporting more LEs. Our hypotheses regarding the aetiology of LEs and depression were only partially supported. We found evidence of genetic influences on independent LEs for both males and females. Dependent LEs were heritable in males but not females. Both independent and dependent LEs were associated with depression symptoms in this study, in line with previous research [1, 13]. However, this overlap was largely explained by non-shared environmental factors, rather than genetic influences, contrary to previous research in different populations which has emphasised the importance of genetic factors in explaining the covariation [21, 22]. This suggests that the relationship between LEs, particularly independent, and depression symptoms are not confounded by genetic influences in this sample.

### Socio-demographic correlates of independent and dependent LEs

The strong association between financial strain and LEs is in line with research in high income countries [2]. This relationship could be due to increased difficulties associated with financial strain such as not being able to afford health care or a healthy diet, and putting a strain on relationships, all of which may make LEs more probable. Although traditionally education is seen as a marker of social economic status [39], we saw little evidence of a relationship between education and either independent or dependent LEs. We found that higher levels of education were associated with lower reporting of dependent LEs. Older age was generally associated with reporting fewer LEs, however, middle age was associated with reporting more dependent LEs. This is largely in line with previous research in HICs which shows that younger age is associated with reporting more LEs [2]. Previous studies have reported mixed findings regarding the prevalence of LEs in males and females [2], in the current sample we found that women were more likely to report independent LEs but less likely than males to report dependent LEs. Being was associated with fewer LEs than those who had been previously married. This may make sense as some of the LEs reflect relationship problems and death of family members. Living in a more urban environment was more protective in terms of experiencing either independent or dependent LEs. This is interesting as the urbanization of LMIC is often seen as a potential risk factor for the development of health disorders [40]. This does not appear to be the case for LEs, a risk factors associated with mental health conditions, in Sri Lanka.

### Aetiology of independent and dependent LEs and depression symptoms

Independent LEs showed low but significant heritability and were mainly influenced by non-shared environment. This finding is contrary to the theory that ‘independent’ LEs are not be influenced by the individual’s behavior. However, a number of other studies have also shown a genetic influence on independent LEs [11]. It is possible that some events that we categorised as ‘independent’ were in fact somewhat ‘dependent’ on the individual’s behaviour. As we were assessing lifetime-ever LEs, it is possible that participants’ current depression symptoms could have affected their recall of independent LEs. Alternatively, research has consistently shown that social economic status has an influence on the number of both independent and dependent LEs. Social economic status has been shown to be heritable in previous samples and, therefore, influence the aetiology of independent LEs [41, 42]. As such, whilst the events are ‘independent’ of the individuals’ direct behaviour, other factors in the environment may influence participants’ likelihood of experiencing them, which would affect their heritability.

Sex differences were identified in the aetiology of dependent LEs, with moderate heritability in males but low heritability in females. This finding is consistent with the moderate heritability found in a previous study using data from the current sample at a previous time point [24]. The heritability in males may be indicative of gene-environment correlation. Higher heritability might be expected in males compared to females as women in Sri Lanka may have less opportunity to select their environment than men. For example, women are often required to defer to men for decision making and are typically limited to conventionally ‘feminine’ jobs [43].

The small but significant contribution of shared environment to the aetiology of dependent LEs is not consistent with studies in Western populations. While this may represent a finding that is specific to Sri Lanka, it may be that twin studies in Western populations have been unable to detect small contributions that shared environment contributions may have due to the low power to detect *C* in the classical twin design. Evidence of non-shared environment contributing to the majority of the variance in experiencing independent and dependent LEs is in line with estimates from Western countries. However, the nature of the environmental exposure may differ between countries.

As seen in previous investigations in Sri Lanka [25], lower heritability of depression symptoms was identified in males, whereas females showed moderate heritability. These results are different to a meta-analysis of twin studies in Western populations which estimated depression heritability at 37%, with 63% of variance explained by environmental factors and found no evidence of sex differences in aetiology [44]. The low heritability in males could be explained by the greater environmental variation in Sri Lanka, compared to Western countries. The higher heritability in females may be accounted for by the low variation in environmental exposures due to cultural gender limitations. Alternatively, it may be that the high variation in environmental exposures in Sri Lanka (e.g. relative poverty) are not causal in female depression symptoms [25]. However, this explanation is not supported by the finding that poverty-related LEs significantly predicted depression symptoms.

### Phenotypic relationship between independent and dependent LEs and depression symptoms

Mean levels of depression symptoms were low but consistent with studies of depression in South Asia [6, 45, 46]. This lower prevalence in South Asian populations could be due to a range of factors including cultural differences in participants’ willingness to disclose symptoms of depression [24, 46]. It is possible that it is also due to differences in the manifestation of depression cross-culturally and, therefore, the relevance of diagnostic criteria or the sensitivity and specificity of symptom questions. Studies have, however, tended to support the validity of diagnostic symptoms [24, 45]. Both independent and dependent LEs were significantly associated with depression in line with previous research [13].

### Genetic and environmental influences on the relationship between LEs and depression symptoms

Sex differences were not observed in the univariate analysis of independent LEs, therefore, the identified sex differences in the independent LEs-depression relationship may be accounted for by sex differences in depression symptom aetiology. In males and females, only non-shared environment significantly contributed to the phenotypic correlation between independent LEs and depression. In males, the majority of the phenotypic correlation between dependent LEs and depression symptoms could be explained by genetic and non-shared environmental influences. In females the relationship between dependent LEs and depression symptoms was explained by non-shared environment. This suggests that the relationship between independent LEs and depression symptoms in Sri Lanka does not appear to be confounded by genetic influences, which put individuals at risk of both experiencing a LE and depression. However, it should be noted that gene-environment interactions between *A* and *E* would be estimated in the *E* component and, therefore, our estimate of non-shared environmental influences may not be entirely independent of genetic influences [37].

### Strengths and limitations

The results need to be viewed in light of several limitations. First, self-reported LEs and depression symptoms may be affected by current or depressed mood of the participants, perhaps inflating the relationship between LEs and depression symptoms. Additionally, certain LEs may have been underreported due to the cultural appropriateness and stigma associated with reporting them e.g. sexual assault. Second, some LE items may have been shared across twin pairs (e.g. ‘family member other than spouse or child dies’). Twin correlations were run excluding items which may have been shared across pairs and results were very similar suggesting this did not affect the results (see appendix eTable 4). Third, whilst co-efficient of reliability for the measures were adequate this reduced reliability may have affected results. Forth, while the BDI-II allowed for the determination of depression symptom severity, it does not provide a clinical diagnosis. Finally, generalisability of results needs to be considered. While the sample is representative of people living in the Colombo District of Sri Lanka, it may not be representative of different regions of Sri Lanka. The inclusion of a singleton cohort is a strength of this study because whilst twins are generally representative of the general population, it allowed us to examine differences in their experiences. We found that singletons reported significantly greater depression scores and numbers of LEs (except for work-related LEs) than twins. This may be suggestive of a protective factor related to being a twin in this population.

## Conclusion

This study investigated the prevalence and underlying aetiology of independent and dependent LEs using data from a representative twin and singleton population study based in Colombo, Sri Lanka. This is the first study to use bivariate twin modelling to investigate the relationship between LEs and depression symptoms in a South Asian population. This study has several implications for future LEs research. Our results suggest similar social-demographic factors are associated with independent and dependent LEs in both Western and South Asian populations. Association between both independent and dependent LEs and depression symptoms were moderate and in line with previous investigations in different cultures. Our finding that the relationship between independent LEs and depression symptoms is largely driven by non-shared environmental influences suggests that enacting policies that reduce individuals’ exposure and increase individual resilience to LEs could result in lower incidence of depression.

## Electronic supplementary material

Below is the link to the electronic supplementary material.

Supplementary material 1 (DOCX 42 kb)
