# Supplementary material for: The relationship between independent and dependent life events and depression symptoms in Sri Lanka: a twin and singleton study
Source: Soc Psychiatry Psychiatr Epidemiol. 2019 Sep 3;55(2):237–49. doi: 10.1007/s00127-019-01765-z (PMC7024056; doi:10.1007/s00127-019-01765-z)
Supplement: Supplementary file 1 — Supplementary material 1 (DOCX 42 kb) [file 127_2019_1765_MOESM1_ESM.docx]

Electronic Appendix

eTable 1: Participating twins in COTASS-2

| Zygosity | Number of individuals | Number of twin families |
| --- | --- | --- |
| MZM | 533 | 295 |
| DZM | 366 | 215 |
| MZF | 730 | 397 |
| DZF | 485 | 281 |
| DZOS | 809 | 467 |

Table adapted from (30). Number of individuals in the twin group is not twice the number of families due to missing data. Number of complete pairs not given as it varies by variable. MZM=monozygotic male, DZM=Dizygotic male, MZF=monozygotic female, DZF=dizygotic female, DZOS=dizygotic opposite sex.

Table e2: Fit statistics for univariate ACE models

|  |  | Model Fit | | | | | |
| --- | --- | --- | --- | --- | --- | --- | --- |
| Measure | Model | -2LL | df | LRT | Δdf | AIC | p |
| Independent life events | Constrained Saturated | 421.18 | 3909 | - | - | -7396.82 | - |
|  | Heterogeneity | 421.22 | 3910 | 0.04 | 1 | -7398.78 | 0.85 |
|  | **Homogeneity*** | **421.41** | **3913** | **0.19** | **3** | **-7404.59** | **0.98** |
| Dependent life events | Constrained Saturated | 2290.09 | 3909 | - | - | -5527.91 | - |
|  | **Heterogeneity *** | **2290.17** | **3910** | **0.08** | **1** | **-5529.83** | **0.77** |
|  | Homogeneity | 2310.45 | 3913 | 20.28 | 3 | -5515.55 | <0.01 |
| Depression | Constrained Saturated | 7302.83 | 3893 | - | - | -483.17 | - |
|  | **Heterogeneity *** | **7303.07** | **3894** | **0.10** | **1** | **-485.06** | **0.74** |
|  | Homogeneity | 7445.48 | 3897 | 142.54 | 3 | -348.52 | <0.01 |

Note: Heterogeneity model was compared to the constrained saturated model. The Homogeneity model was compared to the heterogeneity model. -2LL = negative 2 log likelihood; df = degrees of freedom; LRT = likelihood ratio *X^2^* test comparing the -2LL fit of each model to the -2LL fit of the saturated model; Δdf = difference in degrees of freedom comparing each model to the saturated model; AIC = Akaike’s Information Criterion (lower values reflect a better fit); p = *p*-value. *Best fitting model

Table e3: Fit statistics for bivariate ACE twin models

|  |  | Model Fit | | | | | |
| --- | --- | --- | --- | --- | --- | --- | --- |
| **Measures** | Model | -2LL | df | LRT | Δdf | AIC | p |
| Independent life events-depression | Constrained Saturated | 7318.56 | 7795 | - | - | -8271.64 | - |
|  | Heterogeneity Model* | 7320.41 | 7798 | 2.05 | 3 | -8275.59 | 0.56 |
|  | Homogenetity Model | 7480.76 | 7807 | 160.35 | 9 | -8133.24 | <0.01 |
| Dependent life events-depression | Constrained saturated | 8844.51 | 7795 | - | - | -6745.49 | - |
|  | Heterogeneity Model* | 8855.47 | 7798 | 10.96 | 3 | -6740.53 | 0.01 |
|  | Homogenetity Model | 9037.64 | 7807 | 182.17 | 9 | -6576.36 | <0.01 |

Note: Heterogeneity Model: different magnitude of genetic and environmental influences on variables and covariance between variables by sex. Homogeneity Model: genetic and environmental influences on and across variables equated across males and females. -2LL = negative 2 log likelihood; df = degrees of freedom; LRT = likelihood ratio *X^2^* test comparing the -2LL fit of each model to the -2LL fit of the saturated model; Δdf = difference in degrees of freedom comparing each model to the saturated model; AIC = Akaike’s Information Criterion (lower values reflect a better fit); p = *p*-value. *Best fitting model

Table e4: Twin correlations independent life events, dependent life events excluding possible overlapping events

|  | Independent life events | Dependent life events |
| --- | --- | --- |
| MZM | .28 | .46 |
| DZM | .17 | .21 |
| MZF | .32 | .34 |
| DZF | .20 | .29 |
| DZOS | .10 | .19 |

Note. MZM=monozygotic male, DZM=Dizygotic male, MZF=monozygotic female, DZF=dizygotic female, DZOS=dizygotic opposite sex.
